# Prediction underlying comprehension of human motion: an analysis of Deaf signer and non-signer EEG in response to visual stimuli

**DOI:** 10.3389/fnins.2023.1218510

**Published:** 2023-10-12

**Authors:** Evie A. Malaia, Sean C. Borneman, Joshua D. Borneman, Julia Krebs, Ronnie B. Wilbur

**Affiliations:** ^1^Department of Communicative Disorders, University of Alabama, Tuscaloosa, AL, United States; ^2^Department of Linguistics, Purdue University, West Lafayette, IN, United States; ^3^Linguistics Department, University of Salzburg, Salzburg, Austria; ^4^Centre for Cognitive Neuroscience, University of Salzburg, Salzburg, Austria; ^5^Department of Speech, Language, and Hearing Sciences, Purdue University, West Lafayette, IN, United States

**Keywords:** sign language, predictive processing, entrainment, EEG, optical flow, motion, comprehension

## Abstract

**Introduction:**

Sensory inference and top-down predictive processing, reflected in human neural activity, play a critical role in higher-order cognitive processes, such as language comprehension. However, the neurobiological bases of predictive processing in higher-order cognitive processes are not well-understood.

**Methods:**

This study used electroencephalography (EEG) to track participants' cortical dynamics in response to Austrian Sign Language and reversed sign language videos, measuring neural coherence to optical flow in the visual signal. We then used machine learning to assess entropy-based relevance of specific frequencies and regions of interest to brain state classification accuracy.

**Results:**

EEG features highly relevant for classification were distributed across language processing-related regions in Deaf signers (frontal cortex and left hemisphere), while in non-signers such features were concentrated in visual and spatial processing regions.

**Discussion:**

The results highlight functional significance of predictive processing time windows for sign language comprehension and biological motion processing, and the role of long-term experience (learning) in minimizing prediction error.

## 1. Introduction

The understanding of the human brain as a source of cognition has historically focused on the brain as generating a response to external stimuli. The rapidly-developing field of neuroscience, however, has contributed to a paradigm shift, whereby the traditional concept of the brain as a passive, bottom-up receiver of external information has been replaced by the notion of the brain as an active predictor of the environment based on perceptual inference (Rao and Ballard, 1999; Friston, 2008; Auksztulewicz and Friston, 2015).

In the last decade, the hierarchical generative framework for predictive processing^1^ has been applied to a variety of brain functions, including language production and comprehension (Malaia et al., 2021b; Radošević et al., 2022). However, the neurobiological fundamentals of the bi-directional interface between sensory inference and top-down predictive processing, based on prior learning, remain poorly understood. In this context, neural responses to stimuli can be understood differently depending on the population's experience. This provides an opportunity to refine the understanding of the mechanistic implementation of predictive processing and to identify functional neural dynamics responsible for the computations that support predictive processing. Neuroimaging research has previously demonstrated that neural organization in humans is affected by the structure of the native language (Malaia et al., 2012; Wei et al., 2023). The question that motivated our study was to understand how differing language modality experience may affect predictive processing functions of the brain in the visual domain.

All humans, regardless of sign language experience, are sensitive to visual biological motion stimuli (Bradley et al., 2022; Shen et al., 2023). Human neural response to general (non-linguistic) biological motion is highly robust—sensitivity to it develops regardless of sign language experience (Baldassano et al., 2018), and even in the absence of early visual input (Bottari et al., 2015). Recent MEG work has identified selective cortical tracking of biological motion information, with neural oscillations of observers entraining to the hierarchical kinematic structures of walking sequences at lower-frequency rhythmic structures (Shen et al., 2023). Notably, neural activity of observers in the Shen et al. (2023) study was characterized by enhanced responses to upright stimuli, as compared with neural response to inverted stimuli. Further, the amplitude of the neural coherence response was highest around the temporo-parietal regions of the right hemisphere, known to engage in spatial processing. Thus, all humans possess a general cortical mechanism that encodes low-frequency periodic features of body movements.

However, there is a population that has a unique long-term experience with human motion: users of sign languages. Prior fMRI research into American Sign Language (ASL) processing has indicated that signers rely on motion—i.e., dynamic changes to articulator shape—to understand a message in sign language (Malaia et al., 2012, 2021a; Malaia and Wilbur, 2019; Radošević et al., 2022; Brozdowski and Emmorey, 2023). Additionally, neuroimaging studies in ASL indicated that signers process sign language-related motion differently from non-signers in terms of the topography (distribution among brain regions) of brain activations. While non-signers relied on occipital regions and the temporoparietal junction for motion processing (Malaia et al., 2012), signers processed the same physical differences in the visual signal as containing a meaningful message; engaging anterior and parietal regions of the brain. Notably, the activation processes in signers were highly efficient, indicating minimal cortical engagement in terms of blood oxygen requirements (Malaia et al., 2012, 2014).

These findings complement a body of literature which has established that sign language motion signals as being richer in information content, than non-communicative motion (Malaia et al., 2016, 2022; Borneman et al., 2018). Specifically, sign language communication has higher Shannon entropy,^2^ as compared to non-linguistic human motion (Gurbuz et al., 2020; Malaia et al., 2022). By virtue of containing an information-dense signal, sign language stimuli provide a unique test-bed for the identification of neural bases of predictive processing. The neural dynamics of sign language processing should proceed as biological motion processing in non-signers, and as language processing in signers, thereby revealing neural dynamics specific to each type of computation.

In order to assess the relationship between the neural response to a sign language signal and its time-reversed version in both Deaf^3^ signers and hearing non-signers, we recorded EEG data from participants while they were viewing stimuli videos, which consisted of short sentences in Austrian Sign Language produced by a Deaf fluent signer. The control condition consisted of the same videos in time-reversed format, which preserved spatiotemporal variability of the signal in terms of low-level visual and dynamic features, while rendering the stimulus incomprehensible for sign language users (equivalent to a time-reversed speech signal), and also making it observably atypical in terms of biological motion recognition for non-signing participants. We used cortical coherence as a measure of neural entrainment to visual changes in the videos. Building on the understanding of signers' sensitivity to entropy of the dynamic, information-bearing visual signal (Ford et al., 2021; Malaia et al., 2021a), we tracked the cortical dynamics of comprehension in the visual modality using optical flow measures of the stimuli (Borneman et al., 2018). Elicitation of overt behavioral judgments of stimuli from participants allowed us to have a behavioral control for the higher cognitive task at hand.

Due to the presence of a large number of parameters (62 frequency bins each for amplitude and timing, four regions of interest over the scalp, two populations, and two stimuli conditions), and inherently non-normal distribution of EEG frequency data (Kiebel et al., 2005), we used machine learning, which served as pattern recognition algorithms, in order to assess the salience of specific parameters for brain state classification accuracy. These parameters in-turn, identified the parameters on which population and task-related neural responses differed. Machine learning approaches have been successfully used for brain state classification tasks based on spectral EEG parameters elicited during visual stimulation (Vanegas et al., 2018; Ford et al., 2021). We used a supervised learning paradigm with timing, topography, and amplitude of coherence between EEG and optical flow in visual stimuli as input parameters. This allowed us to make defensible predictions for predictive processing manifestations in higher cognition (language comprehension) task vs. biological motion detection task, while controlling for multiple hypotheses testing. Specifically, based on existing literature (cf. Malaia et al., 2012; Borneman et al., 2018; Blumenthal-Drame and Malaia, 2019; Shen et al., 2023), the following competing hypotheses regarding the neural bases of predictive processing in the two populations can be proposed:

*Hypothesis 1:* Deaf and hearing signers will exhibit similar neural entrainment to the hierarchical (multi-frequency) kinematic structures of sign language/biological motion sequences. Similarity in feature salience across multiple frequency ranges would indicate multi-level spatiotemporal parsing of visual input in either population.
*Hypothesis 2:* Alternatively, increased salience of features in low frequencies differing between populations would suggest learning-based predictive processing patterns correlated with the perceptual sensitivity to visual stimuli as communicative vs. biological motion signals.
*Hypothesis 3:* Spatio-temporal distribution of salient parameters across regions of interest is likely to differ between Deaf signers and hearing non-signers, consistent with the neural bases of language (distributed fronto-temporal network^4^) vs. biological processing (right-lateralized temporo-parietal network) observed in previous studies, indicative of the underlying neural mechanisms.

The hypotheses are partially in conflict (e.g., both Hypotheses 1 and 2 cannot be correct at the same time). This study, thus, aims to bridge the gap in the current literature regarding frequency-specific temporal distribution of neural activity across the gross regions of the scalp during processing of visual information. Together, a combination of temporally-sensitive EEG data and machine learning approaches for investigating cognitive processing dynamics can provide valuable insights into the functional significance of spatiotemporal parameters of brain activity in populations that differ drastically in their visual experience: Deaf signers and hearing non-signers.

## 2. Materials and methods

### 2.1. Participants

Twenty-four Deaf signers and twenty hearing participants (11 Deaf and 16 hearing females) took part in the study. The ages of Deaf participants varied between 28 and 68 years (Deaf *M* = 42, *SD* = 12); hearing participants were between 16 and 31 years old (*M* = 23, *SD* = 4.6). Deaf signers were proficient, everyday users of Austrian Sign Language, according to self-reports provided during the intake interview. The non-signer control group consisted of sign-naive participants who did not know any sign language. All participants reported corrected-to-normal or normal vision, and none had history of neurological disorders. All hearing participants were right-handed (Oldfield, 1971). Five of the Deaf participants were left-handed. All procedures in the study were undertaken with the understanding and written consent of each participant. The Institutional Review Board of the University of Salzburg approved the design of the study as conforming to the Declaration of Helsinki (World Medical Association, 2013).

### 2.2. Stimuli and procedures

Each participant was shown a pseudo-randomized set of videos consisting of two primary conditions and filler videos. Condition 1 included 40 videos of short Austrian Sign Language (ÖGS) sentences (each of which included subject, object, and verb signs), produced by a Deaf signer. Condition 2 was comprised of the same videos as Condition 1, but played in reverse. Other videos of signed sentences (*N* = 200, all containing full signed sentences with varying linguistic structures) were randomly presented among the stimuli for the two conditions of interest. Pseudo-randomization procedure was used to ensure that each condition of interest did not occur more than three times in a row. Four different pseudo-random orders of stimuli were balanced among participants.

Stimuli videos, 1280 × 720 pixels, were presented in the middle of the screen 35.3 × 20 cm in size. The experiment started with task instructions in participant's native languages, and a presentation of a training block of videos. Hearing L1 ÖGS signers were present during the experimental session, and each participant was encouraged to ask clarifying questions regarding the task requirements. Every trial began with a 2-s long presentation of a fixation cross, followed by 200 ms of empty black screen, and a stimulus video (the videos varied in length between 5 and 7 s). After the sentence video was presented, the participant had 3 s to provide the rating, using a keyboard button-press. Deaf participants' task was to rate, on a scale from 1 to 7, whether the stimulus was a good sign language sentence or not (1 stood for “that is not sign language”; 7 stood for “that is good sign language”). Hearing participants rated the video as to whether the video was presented in direct or reverse mode; then, they rated the certainty of their decision on a Likert scale from 1 to 7 (1: very unsure; 4: 50% sure; 7: very sure). Participants could take breaks between presentation blocks (each consisted of 20 videos), but were requested to avoid excessive motion during the presentation of the video material.

### 2.3. EEG acquisition

**Figure 1:**
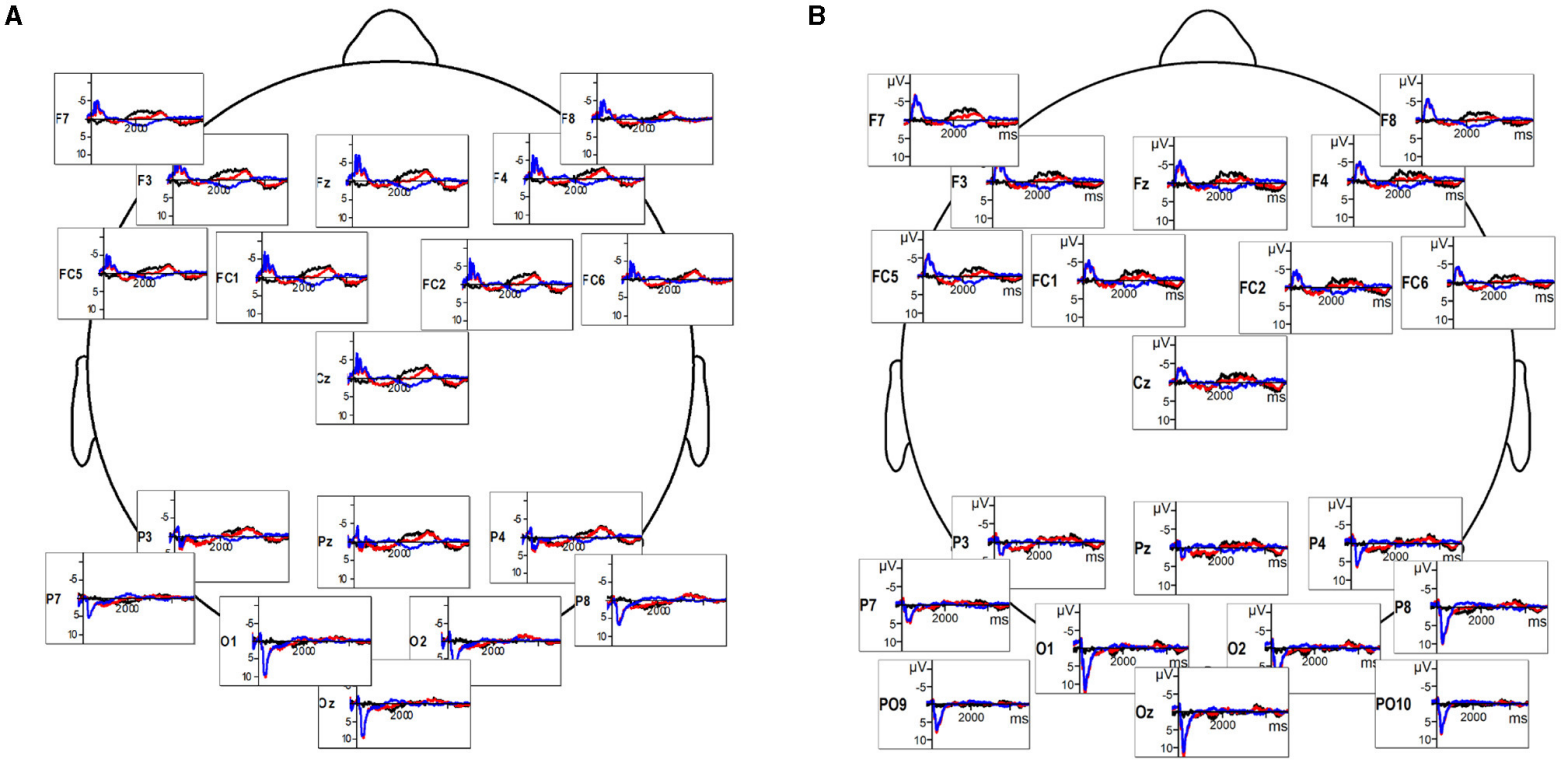
**(A)** Deaf participants' EEG response to sign language videos (red), reversed videos (blue), and the difference wave indicating the timeline of response difference (black). **(B)** Hearing participants' EEG response to sign language videos (red), reversed videos (blue), and the difference wave (black).

Data collection was carried out on a 26-channel active electrode EEG system (Brain Products). The electrodes were placed on the participant's scalp according to the standards of the 10/20 system (Fz, Cz, Pz, Oz, F3/4, F7/8, FC1/2, FC5/6, T7/8, C3/4, CP1/2, CP5/6, P3/7, P4/8, O1/2), and secured with an elastic cap (Easy Cap, Herrsching-Breitbrunn, Germany). The system included two mastoid channels, two Horizontal eye movements (HEOG) electrodes at the lateral ocular muscles, and two vertical eye movements (VEOG) electrodes fixed above and below the left eye. All electrode impedances were kept below 5 kΩ. AFz channel functioned as the ground during the recording, as all other electrodes were referenced to the left mastoid. EEG data was acquired at a rate of 500 Hz. Video stimuli onsets, with individual codes for each video, were time-stamped within the EEG recordings. Offline, EEG data were re-referenced to the average of the left and right mastoid channels, filtered with a bandpass filter (Butterworth Zero Phase Filters; high pass: 0.1 Hz, 48 dB/Oct; low pass: 30 Hz, 48 dB/Oct). The signal was corrected for ocular artifacts by the Gratton and Coles method, and automatically reviewed for other artifacts (such as minimal/maximal amplitude at −75/+75 μV). EEG data were segmented from onsets of video stimuli to 5 s following the onsets. The full duration of video stimuli was between 5 and 7 s; the 5 s cutoff ensured that only neural activity produced during an ongoing video stimuli was analyzed (see Figure 1 for 5 s timescale EEG in both populations).

### 2.4. Optical flow and video-EEG coherence analysis

Optical flow (OF) calculation, used widely in video processing, is the distribution of apparent velocities of objects in an image. To calculate optical flow, a velocity vector (in pixels per frame) is calculated between two adjacent video frames for each pixel, based on the speed of displacement of the image feature identified in that pixel. MathWorks' MATLAB (The MathWorks Inc., 2022) computer vision toolbox optical flow function was used to process stimuli videos by comparing each frame with the prior frame. The output was a matrix of size equal to the input video frame, where each element identified the magnitude of optical flow velocity (pixels per frame) between the two frames for each corresponding pixel in the video. An optical flow histogram (a velocity spectrum) was then created per video frame. The amplitudes across all velocity bins for each frame were summed to calculate global magnitude of optical flow per frame, resulting in optical flow timeseries. For each optical flow velocity timeseries (generated for each stimulus video), coherence between the stimulus video and the neural response in each electrode for each participant was calculated. To compute coherence at a given frequency, both EEG and optical flow timeseries were first filtered at that frequency (from 0.2 to 12.4 Hz, as limited by the duration and Nyquist frequency of the video data) using a second-order IIR bandpass filter. The timeseries correlation was then calculated using canonical component analysis (CCA) with MATLAB NoiseTools toolbox (de Cheveigné et al., 2018). The peak amplitude of the correlation and the timepoint of that correlation were extracted for each frequency, for each electrode, participant, and video.

### 2.5. Data setup and machine learning pipelines

To construct the data matrix, peak cross-correlation time and amplitude values were used for each of the 62 frequency ranges (binned from 0.2 to 12.4 Hz in 0.2 Hz bins). Sensor data were averaged over regions of the scalp (Anterior, Posterior, Left, Right) for each condition and participant. Anterior region included electrodes F3, F4, Fz, FC1, and FC2; posterior region included electrodes P7, P8, P3, P4, Pz; left hemisphere region included electrodes C3, FC5, T7, CP1, CP5; and right hemisphere region included electrode channels C4, FC6, T8, CP2, CP6. This procedure produced a matrix with 496 features (4 regions × 62 frequency bins for peak correlation parameters, and 4 regions × 62 bins for timing parameters) and 88 instances (24 Deaf + 20 control participants' brain responses to two conditions: sign language and reversed video). As differences in feature/instance data distribution, which are especially prominent in human neural data, can negatively impact the performance of machine learning algorithms by over-weighting input parameters, we scaled the data such that each of the parameters would have a mean value of zero and a standard deviation of one (Starovoitov and Golub, 2021).

The processing proceeded in two steps. First, we evaluated the predictive value (i.e., saliency) of input features for each group of participants, and for the stimuli types (i.e., sign language and time-reversed videos). As input features consisted of amplitude and timing of coherence data separated by scalp topography (anterior, posterior, left, right), we aimed to quantify the salience of specific frequency ranges and brain regions for the motion comprehension task in signers and non-signers. We then applied the 10-fold cross-validation approach with the test harness pipeline configuration to prevent data leakage between training and testing data in each cross-validation harness to understand the differences in feature salience for brain state classification across participant groups.

#### 2.5.1. Classification algorithms

Six classifier algorithms were used to evaluate the performance: two linear algorithms [Linear Regression (LR) and Linear Discriminant Analysis (LDA)], and four non-linear algorithms [k-nearest neighbors (kNN), classification and regression trees (CART), Naïve Bayes (NB), and support vector machines (SVM)] from Python *sklearn* library (Pedregosa et al., 2011). Machine learning algorithms, in general, are data-greedy methods that create complex representation models based on raw data. As the algorithms vary in terms of weighting of different parameters of the raw data, it is difficult to predict which types of algorithms will perform well in each particular case, especially on human data, which is highly variable within participant groups. The six classifier algorithms used in the analysis included algorithms that differed in baseline assumptions about the data. For example, linear algorithms [Linear Regression (LR) and Linear Discriminant Analysis (LDA)] assume Gaussian distribution of the data, but differ in terms of performance on well-separated classes (i.e., LR can be less stable than LDA in such cases). Classification and regression trees (CART) are simple pruning algorithms that perform much better than linear algorithms in the presence of outliers. The Naïve Bayes (NB) algorithm assumes conditionally independent (i.e., non-interacting) parameters; although this assumption is unlikely to hold on human data, the algorithm performs well on data sets where parameter dependence is noisy. The K-nearest neighbors (kNN) algorithm performs well where training and testing data sets are very similar (i.e., individual participants' parameters are alike across the population); poor performance on it indicates high variability between participants in the group. The Support vector machines (SVM) algorithm is the most flexible in the sense that it makes no assumptions, but rather learns problem representation from the data; however, it is also the most data-greedy approach. Application of multiple algorithms to the data set, as well as sub-sets (i.e., hearing and Deaf groups separately) can provide valuable insights regarding data distribution, parameter noisiness, and between-participant variability.

#### 2.5.2. Ensemble classifiers

Due to the noisiness of the signal and consequent potential for model overfitting (e.g., by CART and SVM algorithms), we used four ensemble classifiers to reduce the likelihood of such overfitting. The two bootstrap aggregation ensembles, Random Forest (RF) with 100 estimators, and Extra Trees (ET) with 100 estimators, trained multiple models based on each sample, outputting prediction as the average across the difference models. This approach improves sensitivity of decision trees by limiting the number of features used for optimizing each tree. The two boosting ensembles, AdaBoost Classifier (AB) with 50 estimators, and Gradient Boosting Machines (GBM) with 100 estimators, generated multiple models of the data transformation function, each of which attempted to fix the mistakes of the previous models. During classification, the data of 20 percent of each group's participants was retained for validation as a hold-out set. We used 10-fold cross-validation with the test harness pipeline configuration to prevent data leakage between training and testing data in each cross-validation harness.

## 3. Results

### 3.1. Behavioral results

Behavioral data confirmed that Deaf participants' did not consider time-reversed videos of sign language sentences linguistically acceptable (*M* = 1.72, *SD* = 0.76, where response 1 denoted stimuli that were not acceptable as Austrian Sign Language, and 7 denoted clear communication in sign language on a 7-point Likert scale). Austrian Sign language videos, on the other hand, were rated as linguistically acceptable by Deaf participants (*M* = 5.8, *SD* = 1.05). Behavioral data from hearing participants indicated that they were able to identify the direction of the sign language video (normal vs. reversed) with high accuracy (94% for direct videos with mean certainty rating of 4.93, and 84% for reversed videos also with mean certainty rating of 4.93). Thus, the behavioral data confirmed that Deaf signers correctly identified signed sentences as linguistically comprehensible and acceptable, while non-signers were also able to identify the differences between direct and reversed videos with high accuracy and certainty, although they were not able to understand the sentences signed.

### 3.2. Deaf signer data: feature analysis

Information-based Univariate Feature Selection (UFS, see Solorio-Fernández et al., 2020) was used to identify the relevance of input parameters for classification. In general, USF uses a measure of entropy of similarities between features, thus ranking them for the information-theoretic contribution. Posterior, left, and frontal posterior regions correlations between EEG and optical flow in the video at 0.8-1 Hz, and right-hemisphere correlation at 1-1.2 Hz ranked highly, indicating distributed regional contributions to sign language comprehension over a ~1 s time window (see Table 1). Left-hemisphere feature salience was ranked higher than that of the right hemisphere. This appeared to indicate that linguistic processing (characterized by left-hemisphere activity), rather than spatial processing (characterized, for visual stimuli, by right-hemisphere processing) was relevant for signer identification of natural vs. reversed sign language stimuli.

**Table 1:** Top 4 features, as identified by UFS, from both time and correlation amplitude data; correlation amplitude is significant, unless time is noted.

**Dataset**	**Region of interest**	**Frequency bin (Hz)**
Deaf	Posterior	0.8–1.0
Deaf	Left	0.8–1.0
Deaf	Frontal	0.8–1.0
Deaf	Right	1.0–1.2
Hearing	Posterior	0.8–1.0
Hearing	Frontal	0.8–1.0
Hearing	Right	1.0–1.2
Hearing	Left	0.8–1.0
Deaf and hearing	Posterior	1.0–1.2
Deaf and hearing	Frontal	0.8–1.0
Deaf and hearing	Left	0.8–1.0
Deaf and hearing	Left	1.0–1.2
Deaf and hearing (direct only)	Posterior time	7.6–7.8
Deaf and hearing (direct only)	Posterior	0.2–0.4
Deaf and hearing (direct only)	Frontal time	9.4–9.6
Deaf and hearing (direct only)	Posterior	1.8–2.0

**Figure 2:**
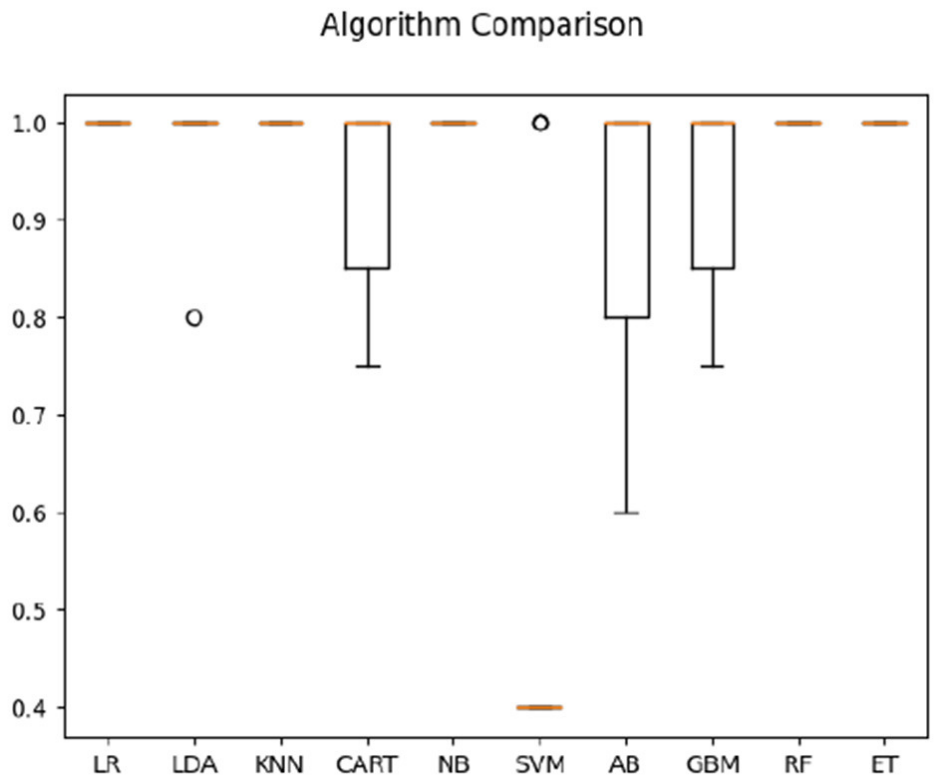
Classification accuracy across algorithms based on Deaf dataset.

In classification of Deaf signer data based on both frequency correlation and timeshift peaks, most algorithms (LR, KNN, CART, NB, RF, and ET) yielded 100% accuracy (see Figure 2), with SVM performing the worst at 52%. For time-parameter analysis, Random Trees algorithm yielded 91.5% accuracy on time data only; timeshifts for frequency coherence bins between 0.2 and 0.4 Hz (posterior, anterior, and left), as well as left-hemisphere activity at 0.6–0.8 Hz frequency were ranked of high importance to classification. Here, the presence of two salient feature bins over the left hemisphere is of note, as it might indicate multi-scale processing of sign language (cf. Blumenthal-Drame and Malaia, 2019).

### 3.3. Hearing non-signers data: feature analysis

**Figure 3:**
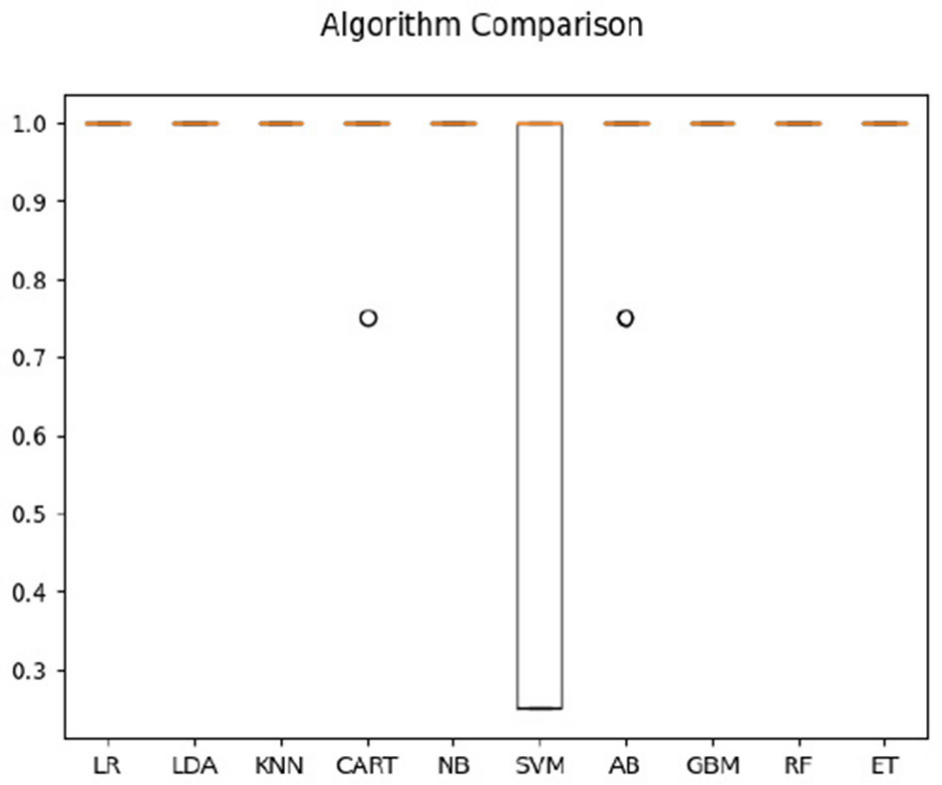
Classification accuracy across algorithms based on hearing participants' data regarding the timing and amplitude of correlations between optical flow and EEG.

**Figure 4:**
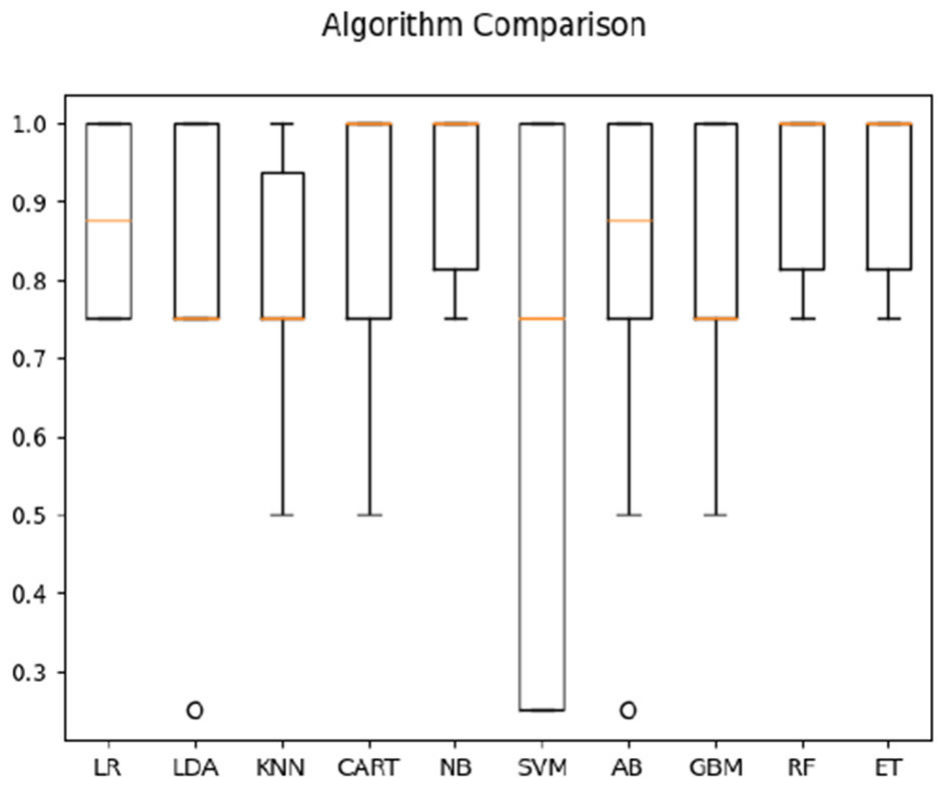
Classification accuracy across algorithms based on hearing participants' data regarding the timing of correlations between optical flow and EEG.

For hearing non-signer data, Univariate Feature Selection (UFS) indicated that posterior, left, and frontal frequency correlations at 0.8 and 1 Hz, as well as right frequency correlations at 1–1.2 Hz were identified as highest-ranked features, highlighting entropy-based contribution of these parameters to the classification task. Notably, left hemisphere features (either time or frequency correlation) did not rank highly for hearing participants. The order of parameter salience, however, differed from that of Deaf-only data, in that right hemisphere correlation ranked higher than left hemisphere. In classification of hearing non-signer data based on both frequency correlation and timeshift peaks, most algorithms (LR, LDA, KNN, NB, GBM, RF, and ET) yielded 100% accuracy, where SVM performed the worst at 70% (see Figure 3). For time-parameter analysis in non-signers, NB, ET, and GBM yielded 92.5% accuracy (see Figure 4). Timeshifts for 0.2–0.4 Hz left, posterior, and anterior coherence was ranked of high importance to classification, as well as 0.6–0.8 Hz coherence over the right hemisphere.

### 3.4. Combined classification results

Using information-based Univariate Feature Selection (UFS), we assessed the relevance of input parameters for classification between the combined Deaf signer and hearing non-signer groups for both conditions (direct and reversed videos). Information-based UFS scores the contribution of each feature based on the entropy of the features using a leave-one-out strategy. Thus, the importance of a particular feature depends on the increase in the value of the entropy of the dataset calculated without that particular feature. In this way, features were ranked from the most relevant to the least relevant. The largest feature-based difference between the two groups was driven by the amplitude of 0.8–1 Hz anterior and 1–1.2 Hz posterior correlations, as well as left-hemisphere correlations between 0.8 and 1.2 Hz (encompassing two frequency bins). This, combined with classification data from each individual population, indicated that entrainment to the stimuli was very strong in both populations, but the topography of entrainment differed between groups.

**Figure 5:**
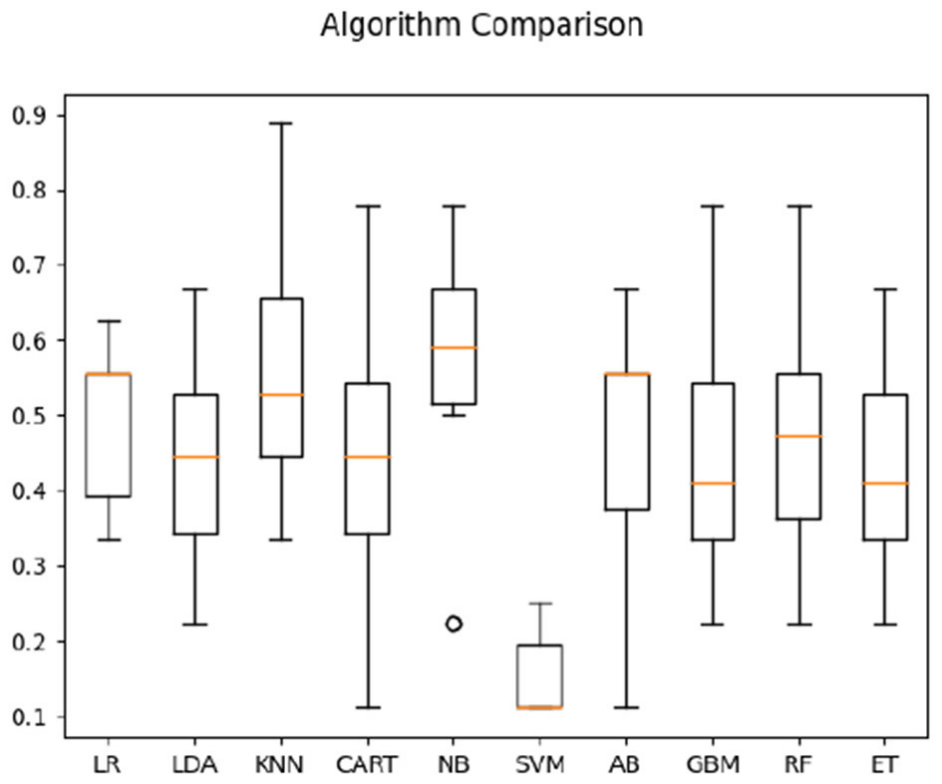
Classification accuracy across algorithms based on both Deaf and hearing participants' data.

Using both the frequency correlation and temporal parameters on the two populations (with four classes; i.e., (1) comprehensible sign language for Deaf signers, (2) meaningless signing for Deaf signers, (3) direct video of sign language not comprehensible by hearing non-signers, and (4) reversed video not comprehensible to hearing non-signers), the highest average classification accuracy was obtained by Naive Bayes algorithm (56%, compared to 25% pure chance—see Figure 5).

**Figure 6:**
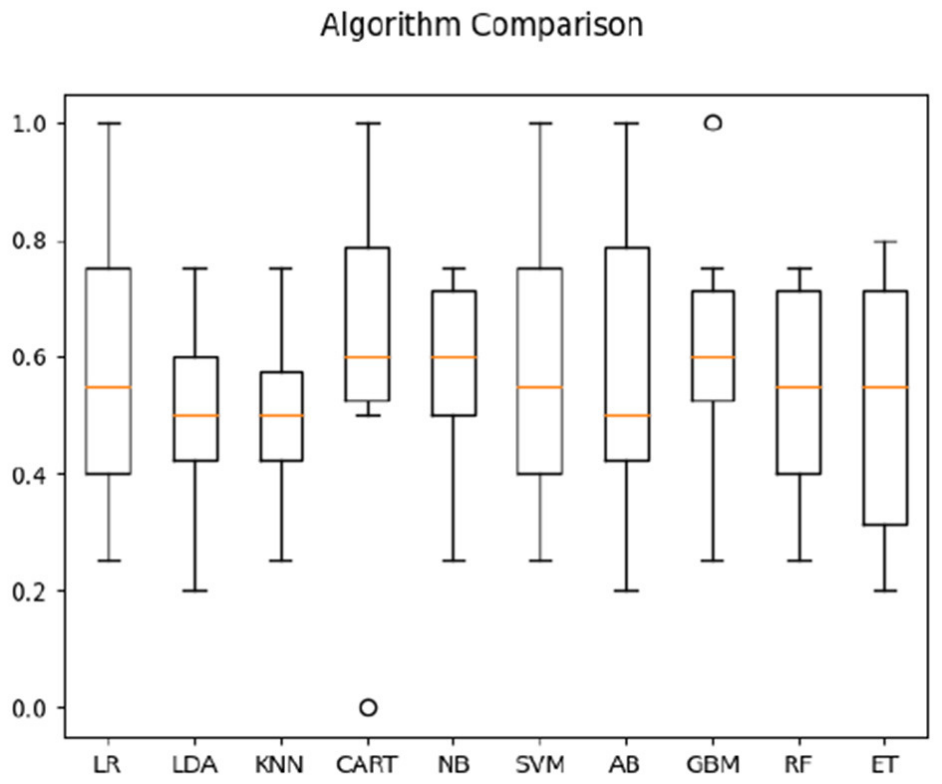
Classification accuracy across algorithms based on both Deaf and hearing participants' data in response to sign language stimuli only.

**Figure 7:**
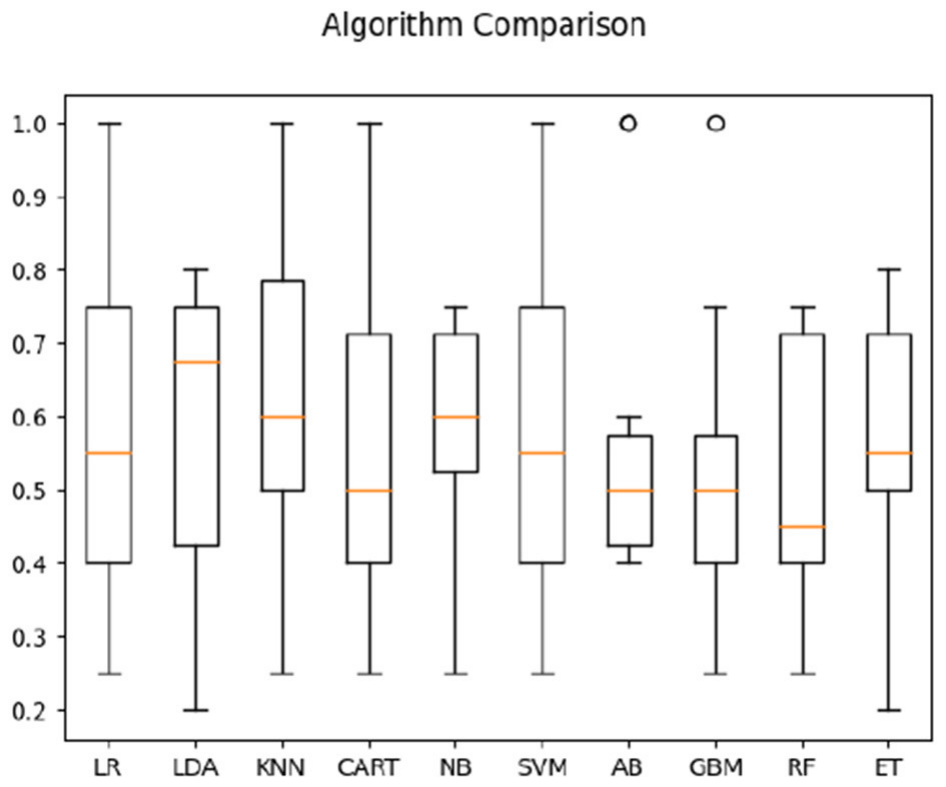
Classification accuracy across algorithms based on both Deaf and hearing participants' time parameters in response to sign language stimuli only.

We then focused on classifying the populations in one condition only—when watching direct sign language videos (comprehensible to signers, but not to non-signers). For both correlation and timeshift data in the direct-video condition for signers and non-signers, highest average classification accuracy (see Figure 6) was obtained by CART (63%). For timeshift data only, the accuracy of the KNN algorithm appeared the highest at 64% (see Figure 7). Interestingly, the features of relevance for this classification were anterior bins at 9.2–9.46 Hz frequencies—i.e., those within the alpha-rhythm of the human brain (typically falling between 8 and 12 Hz). This appeared to indicate that the differences in comprehension vs. non-comprehension of language manifest in the relatively different effort involved in executive processing by signers and non-signers, as noted in the literature previously (cf. Malaia and Wilbur, 2020).

#### 3.4.1. Review of time-based feature analysis and classification

In both populations, peak coherence between the stimuli and neural activity was observed between 100 and 300 ms post-stimulus onset (this was in response to both time-direct and time-reversed stimuli), which is a generally expected time window for visual meaning extraction (Greene and Hansen, 2020).

As noted in Table 2, which presents the top 4 features identified by UFS as salient for classification, both Deaf and hearing participants' data, when considered within each population, indicated low-frequency (i.e., longer temporal window) relevance for identifying direct vs. reversed videos. However, Deaf signers appeared to use multi-scale response of left hemisphere, while hearing participants demonstrated more distributed activity in terms of time-response. When only neural responses to sign language were considered in both populations (without the data in response to reversed videos), the time of frontal responses in the alpha frequency range (9.2–9.6 Hz), as well as left hemisphere responses to 10–10.2 Hz stimuli appeared more critical, indicating executive control engagement for language processing in Deaf participants, but in not hearing controls.

**Table 2:** Top 4 features, as identified by UFS, from the time data only (by dataset).

**Dataset**	**Region of interest**	**Frequency bin (Hz)**
Deaf	Left	0.2–0.4
Deaf	Right	0.2–0.4
Deaf	Frontal	0.2–0.4
Deaf	Left	0.6–0.8
Hearing	Left	0.2–0.4
Hearing	Right	0.6–0.8
Hearing	Frontal	0.2–0.4
Hearing	Posterior	0.2–0.4
Deaf and hearing	Left	0.2–0.4
Deaf and hearing	Right	0.2–0.4
Deaf and hearing	Frontal	0.2–0.4
Deaf and hearing	Posterior	0.2–0.4
Deaf and hearing (direct only)	Left	10–10.2
Deaf and hearing (direct only)	Frontal	9.4–9.6
Deaf and hearing (direct only)	Posterior	2.0–2.2
Deaf and hearing (direct only)	Frontal	9.2–9.4

## 4. Discussion

In the present study, we assessed the relationship between neural responses and the visual stimuli eliciting those responses in Deaf signers and hearing non-signers. The stimuli differed in their likelihood of eliciting conscious processing: the Deaf signers group could recognize and comprehend sign language, while the hearing non-signers could not. The reversed videos, although equivalent in low-level contrast and size (visual frequency) features, made predictive processing impossible for either group—either on the basis of sign language, or on the basis of human motion detection. Behavioral data confirmed that Deaf participants engaged in online comprehension of sign language videos, but did not consider time-reversed videos understandable. Non-signers' behavioral data indicated that although they could differentiate between direct and reversed videos, they could not comprehend either.

To characterize the frequency-following response in the neural data to the sign language signal, we quantified motion-based changes in the video signal using optical flow measured across visual frequencies. This measure was linearly regressed against individual EEG signals of each participant, such that peak cross-correlation frequency, and the timing of the peak, was defined for each channel in the EEG data. The channels were then grouped by topography on the scalp (encompassing anterior, posterior, left, and right brain areas). We used entropy-based feature ranking and a variety of machine learning pipelines to evaluate EEG response parameters that characterized visual comprehension (in signers and non-signers). Independent and joint analyses of frequency-binned entrainment amplitude and timing across the four distinct scalp regions in both populations indicated that brain responses to direct vs. reversed videos were distinguishable based primarily on low-frequency data, suggesting that both the comprehension of sign language and the identification of natural motion relied on convergence of sensory input (bottom-up) and predictive (top-down) processing.

UFS algorithm analyses indicated increased salience of low-frequency parameters within each population separately. These findings supported the proposed *Hypothesis 2* over *Hypothesis 1*. While *Hypothesis 1* proposed similar feature salience between the two populations due to biological motion sensitivity, *Hypothesis 2* suggested that visual experience with a sign language in Deaf signers would result in different spatiotemporal predictive patterns. The results supported the model of predictive processing, which suggest that experience-based patterns correlated with the perceptual sensitivity to visual stimuli as communicative vs. biological motion signals in Deaf signers and hearing non-signers, respectively. Additionally, classification of two-population data yielded feature salience patterns that differed substantially from single-population analyses, supporting *Hypothesis 3*: the distribution of features relevant for between-population classification highlighted the differences between neural bases of sign language processes (distributed fronto-temporal network) vs. biological motion processing (right-lateralized temporo-parietal network).

Another important finding was that both populations showed predictive processing of the motion in the stimuli, in agreement with prior research on biological motion (Shen et al., 2023) and on American Sign Language (ASL) (Brozdowski and Emmorey, 2023). While the prevalence of low-frequency (longer time window) prediction was reasonably expected for signers, who use visual modality as primary means of communication, non-signer familiarity with human gesturing in a non-verbal component of everyday communication also allowed them to also be reasonably accurate in predicting/modeling hand and head motion in video in the stimuli, although at somewhat higher frequencies (i.e., for shorter time windows). The differences between the two populations in the processing of motion were also evident in the topographic distribution of high-ranking features. Deaf signers demonstrated a more equitable distribution of feature relevance across brain regions typically engaged in sign language processing: occipital cortex (visual processing), frontal cortex (executive processing), and left hemisphere (syntactic/semantic processing (cf. Malaia et al., 2021a). Feature relevance distribution in non-signers, on the other hand, indicated relevance of occipital (visual processing) and right-hemisphere (spatial processing) regions for response to visual stimuli. Prevalence of lower frequencies among features of relevance in non-signer data also indicates that processing of visual information on human motion is based on convergence, at the neural level, of predictions and observations. The fact that brain state classification was highly (97%) successful among non-signers also indicated that non-signers engaged in predictive processing, albeit for shorter time intervals than signers, as might be expected for motor vs. linguistic processing (Blumenthal-Drame and Malaia, 2019).

The findings can also be interpreted in the context of Friston (2018) free energy framework, which suggests that, once the learning has taken place, the neuronal dynamics of both sensing and prediction would be optimized for the same cost function. Both the proficient Deaf signers and hearing non-signers engaged in predictive processing, with strong frequency correlations to the optical flow in the stimuli. However, the features of high relevance to classification differed in both topographic and frequency distribution, indicative of predictive processing for different purposes: sign language comprehension for Deaf signers, and prediction/interpretation of natural human motion, for hearing non-signers. This suggests that both populations are optimizing the neuronal dynamics and functional connectivity to enhance neural representations of salient sensory parameters in time and space. The prevalence of lower frequencies among features of relevance in both groups may reflect the brain's ability to select newsworthy prediction errors. The study also highlights the importance of experience-dependent learning for the purposes of predictive processing: the substantial processing differences between the groups were grounded in life-long experience with sign language communication for Deaf signers, and human motion (both gesture and goal-directed motion) for hearing non-signers.

### 4.1. Limitations and further research

Although both populations appeared to use the bottom-up (sensory) and top-down (predictive) processing, the frequencies that were most salient for classification, as well as the topographic distribution of relevant parameters, differed substantially between Deaf signers and hearing non-signers—this is what allowed for the detection of sign language comprehension with high fidelity in a small number of participants. The participants were not matched for age, since the question concerned, primarily, the variability due to sign language ability. The older age of the Deaf signers group might have led to attenuated amplitude of response to the stimuli in the time domain (cf. Moran et al., 2014); however the present analysis focused on the spectral domain. Conducting an equivalent study on the populations in measurable transitional states, such as children acquiring sign language, or adults learning sign language, can provide a more comprehensive understanding of the neural mechanisms underlying the feedback- and feed-forward loops between sensory perception, or inference, and top-down prediction grounded in learning, and changes in the neural bases of these mechanisms over time. For example, in young children, the study can examine how the neural response to sign language stimuli evolves as they learn and acquire the language, and help trace the developmental trajectory for the neural networks involved in language processing as grounded in sensory and cognitive experiences. Since experience plays a significant role in shaping neural networks for language processing, similar analyses of neural data from adult learners of sign language may help characterize the changes in organization of functional neural responses to sign language visual stimuli as learners progress in their proficiency from novice to proficient signers. This can provide insights into the neural plasticity, and the changes in relative weighting of sensory vs. predictive streams as processing shifts from motion recognition to language comprehension. Additionally, studies examining the effects of individual differences (such as sign language proficiency or age of sign language acquisition) on the neural bases of predictive processing may help further specify language- and modality-specific effects of brain organization on predictive processing mechanisms.

## 5. Conclusion

The results for Deaf signers and hearing non-signers show that both populations exhibited coherence to visual stimuli with spectrotemporal parameters of sign language/human motion, indicating engagement in predictive processing. Feature analysis in Deaf signers' data showed a distributed topography of relevant features weighted toward low frequency bins; the same analysis in non-signer data indicated relevance of visual and right-hemisphere regions in processing of the visual-spatial human motion; somewhat higher frequencies of relevance indicated a shorter prediction window for human motion prediction as compared to Deaf signers. The prevalence of lower frequencies among features of relevance suggests that comprehension of visual information is based on convergence, at the neural level, between bottom-up observations and top-down predictions, the latter being the result of prior experience, or learning.

## Data availability statement

The original contributions presented in the study are included in the article/supplementary material, further inquiries can be directed to the corresponding author.

## Ethics statement

The studies involving humans were approved by University of Salzburg review board. The studies were conducted in accordance with the local legislation and institutional requirements. The participants provided their written informed consent to participate in this study.

## Author contributions

EM: conceptualization, methodology, analysis, and writing—original draft. SB: software, analysis, and writing—original draft. JB: software, analysis, and writing—review and editing. JK: conceptualization, data collection, visualization, and writing—review and editing. RW: conceptualization and writing—review and editing. All authors contributed to the article and approved the submitted version.

## Footnotes

^1^Predictive processing of a language-related signal is a neural encoding strategy for linguistic input, which partially pre-generates the expected features of an input signal (thus, convergent input does not need to be encoded/is ignored), and highlights the unexpected features (cf. Bornkessel-Schlesewsky et al., 2022). This is an automatic (i.e., not consciously controlled) process (cf. Friston, 2008).

^2^Shannon (1948) entropy is a quantitative measure of the amount of information contained in a communicative signal, which is related to its predictability. For example, in a sequence of numbers that consists only of 1 and 0 s, the likelihood of each new number is (in the absence of other patterns) 50%. In the visual domain, the metric can be used to quantify spatial and temporal predictability of the signal.

^3^Per convention Deaf with upper-case D refers to deaf or hard of hearing humans who define themselves as members of a sign language community. In contrast, deaf refers to audiological status.

^4^While the fronto-temporal language network's association with language is modality-independent, the use of signed stimuli which are not comprehensible for non-signers means this population is not expected to engage this network in the present study.
